# Differences in infant feeding practices between Indian-born mothers and Australian-born mothers living in Australia: a cross-sectional study

**DOI:** 10.1186/s12889-022-13228-3

**Published:** 2022-05-10

**Authors:** Chitra Tulpule, Miaobing Zheng, Karen J. Campbell, Kristy A. Bolton

**Affiliations:** 1 School of Exercise and Nutrition Sciences, Deakin University, Burwood, Australia; 2 Institute for Physical Activity and Nutrition (IPAN), School of Exercise and Nutrition Sciences, Deakin University, Geelong, Australia

**Keywords:** Infant feeding, Ethnicity, Indian-born mothers, Breastfeeding, Immigrants, Childhood obesity, Complementary feeding, Formula feeding

## Abstract

**Background:**

Immigrant children from low- and middle-income countries (e.g. India) have higher obesity rates than children from high-income countries (e.g. Australia). Infant feeding practices are a key modifiable risk factor to prevent childhood obesity. This study compared infant feeding practices such as breastfeeding, infant formula feeding, timing of introduction to other liquids and solids of Indian-born versus Australian-born mothers living in Australia.

**Methods:**

Data of children aged between 0–24 months from the 2010–2011 Australian National Infant Feeding Survey were analysed. Infant feeding practices between Indian-born mothers (*n* = 501) and Australian-born mothers (*n* = 510) were compared. Multiple regression models with adjustments for covariates, such as maternal demographic factors, were conducted.

**Results:**

Compared to infants of Australian-born mothers, infants of Indian-born mothers were breastfed for 2.1 months longer, introduced solids 0.6 months later and water 0.4 months later (*p* < 0.001). Moreover, infants of Indian-born mothers were 2.7 times more likely to be currently breastfeeding, 70% less likely to currently consume solids and 67% less likely to consume solids before six months (*p* < 0.001). In contrast, infants of Indian-born mothers were introduced to fruit juice 2.4 months earlier, water-based drinks 2.8 months earlier and cow’s milk 2.0 months earlier than infants of Australian-born mothers (*p* < 0.001). Additionally, infants of Indian-born mothers were 2.7 times more likely to consume fruit juice (*p* < 0.001) than the infants of Australian-born mothers.

**Conclusion:**

Significant differences exist in infant feeding practices of Indian-born and Australian-born mothers (some health promoting and some potentially obesogenic). The evidence of early introduction of sweetened fluids in infants of Indian-born mothers provides an opportunity to support parents to delay introduction to promote optimal infant growth..

## Background

Overweight and obesity rates in Australian children is high and is associated with increasing healthcare costs. Importantly the prevalence overweight and obesity varies across socioeconomic and ethnic groups. Recent estimates in 2018–19 suggest that nearly one-third of Australia’s population were born overseas; and 2.4% of the total Australian population comprised of Indian immigrants making them the third largest immigrant population [1, 2]. Immigrant children from low- and middle-income countries (a classification that includes India) have a higher risk of overweight/obesity than children of Australian-born mothers or children of mothers from high-income countries [3]. Zulfikar et.al, reported that children born to mothers from low- to middle-income countries were 50–70% more likely to be overweight or obese [3], increasing the risk of developing type 2 diabetes, elevated triglycerides, blood pressure, and cardiovascular disease in adulthood [4]. The prevalence of overweight and obesity in Australian children and adolescents between the ages of 2–17 years is high (25%) [5], resulting in healthcare costs of approximately AUD 43 million in 2015 [6].

One key modifiable risk factor for developing childhood overweight or obesity is dietary intake [7]. The World Health Organization (WHO) has stressed the importance of the first 1000 days of life (from conception to the first two years after birth) as a critical window for nutritional intervention to reduce the risk of overweight or obesity [8]. A recent review of childhood obesity prevention interventions highlighted the programming effect of early nutrition, including infant feeding practices in obesity development [9].

To support optimal infant nutrition, the WHO, Australian Dietary Guidelines and Indian Infant and Young Child Feeding Guidelines (IYCF) recommend exclusive breastfeeding for the first six months followed by the introduction of solids at around six months of age [10–12]. The evidence-based guidelines highlight the short-term and long-term benefits of breastfeeding for infants, such as slower weight gain in childhood and adolescence and lower obesity risk in adulthood [13]. Early introduction of solids (before the age of four months) and infant formula may contribute to excessive consumption of calories and protein that may be a risk factor for the development of overweight and obesity [14, 15].

Given the higher risk of overweight/obesity in Indian immigrant children, and the potential association of early feeding to child adiposity, it is crucial to understand infant feeding practices in Indian immigrant mothers to promote best-practice [3]. International studies have shown that infant feeding practices among ethnic mothers (including those with an Indian background) are influenced heavily by culture, socioeconomic status, family beliefs, support from family and friends, maternal age and acculturation and the influence of grandparents [16–19].

Ethnic differences in infant feeding practices such as breastfeeding, pre-lacteal feeding, formula feeding, exposure to other liquids (cow’s milk, fruit juice, cordials, teas) and exposure to solids have been documented previously [20–22]. Different cultural beliefs are likely drive these ethnic differences in infant feeding. For instance, studies have shown that colostrum is perceived as harmful and pre-lacteal feeds right after birth (honey, jaggery, cow’s milk) are perceived beneficial in Indian culture, which doesn't align with infant feeding guidelines [21–23]. Few studies examine first foods offered to infants by ethnicity. However, emerging studies have shown that ethnic mothers (including Indian-born) are more likely to introduce sweet foods, cereals, fruits, juice, vegetables and some cases, rice and lentil-based foods as the first foods instead of single cereal foods as recommended by the infant feeding guidelines [12, 22, 24].

The current literature examining infant feeding practices of Indian-born mothers in Australia is minimal [21]. Given the potential for higher prevalence of obesity in the Indian-immigrant population; an improved understanding of infant feeding practices of this specific ethnic group in Australia is warranted. Knowledge regarding infant feeding practices will allow health professionals, and policymakers to design and endorse tailored intervention programs for the Indian community living in Australia that aim to to promote optimal infant feeding behaviours.

Therefore, this study aimed to compare infant feeding practices such as breastfeeding, infant formula feeding, timing of introduction of complementary feeding (feeding other liquids and solids) of Indian-born versus Australian-born mothers living in Australia.

## Methods

### Study design and participants

**Fig. 1:**
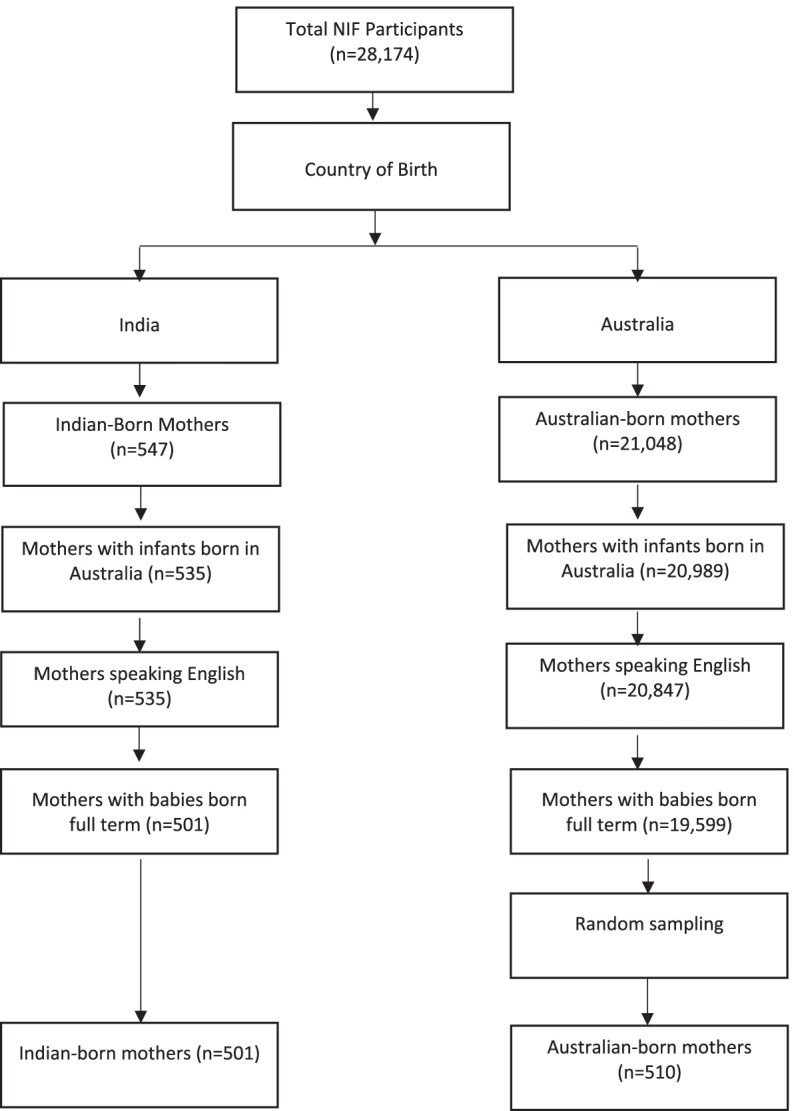
Flowchart of final sampling of Indian-born and Australian-born mothers. *ANIFS* Australian National Infant Feeding Survey [25]

Data from the cross-sectional Australian National Infant Feeding Survey (ANIFS) [25], which captured infant feeding practices and behaviours of infants aged 0–24 months, was analysed [25]. The survey was conducted during 2010–2011 in Australia [25]. Children from 0–24 months were randomly selected nationwide from the Australian Medicare enrolment database [25]. The sampling methodology has been previously described in detail. Briefly, the survey strategy oversampled infants at each month of age up to six months to obtain quality estimates of breastfeeding intensity and duration for this age period [25]. A total sample of 28,759 mothers completed ANIFS (response rate = 56%) [25]. The survey design was piloted on 1000 randomly selected children from Medicare Australian enrolment database to assess ease of understanding and the overall integrity and reliability of the survey [25]. The final questionnaire consisted of 101 questions; 33 questions were used for the present study [25].Fig. 1 presents a flow chart of the final sample analysed. Mothers were included in the analysis if they were either born in India or Australia. Mothers were excluded from the analysis if the infant was born overseas (2% of Indian-born and 0.2% of Australian-born mothers), the Australian-born mother did not speak English at home (0.7%) and if the infant was premature (i.e. born < 37 weeks), 6% each for Indian and Australian-born mothers). Given the extremely large sample of Australian-born mothers; a random sample of Australian-born mothers that was the same size as the Indian-born cohort (*n* = 501) was selected using a random sample command in StataIC 15.0 (Texas, USA), thus minimising selection bias.

### Sample characteristics

Demographic information, including mother/infants’ date of birth and country of birth, infant’s birth weight and length and infant's age in months, were collected. Mothers reported their date of birth, postcode, the main language spoken at home, marital status, maternal educational level, maternal smoking status during pregnancy, parity, total gross household income, maternal weight and height after pregnancy. Pre-pregnancy maternal BMI was calculated [25]. Socioeconomic Indexes For Areas (SEIFA) score of relative disadvantage quintile based on educational qualifications, employment status, marital status, parity and competency in English was used as a proxy for socioeconomic status and lowest quintile representing most disadvantaged [26]. Maternal ethnicity was determined by maternal country of birth (i.e., India or Australia). Indian-born mothers were defined as born in India and now living in Australia [25].

### Measurement of infant feeding practices

Mothers or carers reported infant feeding practices. Ages when breastfeeding, formula feeding and complementary feeding (months) were examined. Mothers also reported current breastfeeding (yes/no) at the time of survey completion, whether infant ever had breastmilk, formula, toddler milk, cow's milk, soymilk, water, water-based drinks, fruit juice (yes/no), and ages when exposed to these drinks. Details of fluids and drinks consumed by infants as described previously [25]. Water-based drinks includes cordial, soft drinks (non-alcoholic, carbonated/ non-carbonated, consisting of artificial colours, flavours and sugar), tea; cow’s milk includes any sips of this milk, flavoured and powdered milk but excludes these kinds of milk combined with solids (cereal); soft, semisolid and solid foods include custards, mashed and foods diluted with water, milk and other fluids; water includes any sips of water but excludes water combined with any other liquids such as cordial (a sweet non-alcoholic drink made from fruit juice) or solids (formula).

### Statistical analysis

Descriptive statistics were conducted (mean ± SD or proportion) to summarise sample characteristics and infant feeding practices. The predictor (independent) variable in all analyses was ethnicity; and the outcome (dependent) variables were the infant feeding practices. Chi-square and t-tests were used to test categorical and continuous infant feeding practices between ethnic groups (Indian-born and Australian-born). Pearson’s correlation coefficients matrices assessed multicollinearity amongst all independent variables (ethnicity and covariates) in the adjusted models; and no evidence of multicollinearity was found [27], Multiple linear regression examined the association between ethnicity and continuous outcome variables such as age in months, when the infant stopped receiving breastmilk, age in months when exposed to infant formula, and other liquids and solids. Binary logistic regressions tested the association between ethnicity and dichotomous outcome variables (yes/no) such as ever had breastmilk, cow's milk, toddler milk, soymilk, formula, water, water-based drinks, fruit juice and soft semisolid/ solid foods) and dichotomous age of ever stopped breastfeeding, introduction water-based drinks, soft semisolid/solid foods and introduction to fruit juice (< six months and ≥ six months). Ages in months when introduced water-based drinks, fruit juice and solids were examined by dichotomising the infant's age into < four months and ≥ four months and < six months and ≥ six months, in alignment with infant feeding guidelines [12]. Due to a small number of infants in the < four months group, a six month cut off was used for variables such as age when first exposed to water-based drinks, soymilk, fruit juice, age when had semisolid/solid foods and age when stopped breastfeeding.

Each infant feeding variable was assessed in separate models with an unadjusted and an adjusted model. The unadjusted model (model 1) included ethnicity (Indian-born and Australian-born) as the predictor variable nd each infant feeding practice as the outcome variable. The adjusted model (model 2) included the following covariates: infant’s age at the time of survey completion, maternal age, pre-pregnancy BMI and area level of disadvantage (SEIFA). The present study used pre-pregnancy maternal BMI in the regressions as previous studies have shown that higher pre-pregnancy maternal BMI increased the odds of children being obese in later life [28]. Variables such as gross household income, presence of spouse post-birth, maternal educational qualifications were not included in the adjusted model as they were part of SEIFA. All analyses were conducted using IBM SPSS Statistics v26.0. (IBM Corp, Armonk, NY), with a significance level set at *p* < 0.05 (two-sided).

## Results

### Maternal demographic characteristics

There were several differences in demographics between Indian-born and Australian-born mothers, as shown in Table 1. Compared to Australian-born mothers, Indian-born mothers had a lower mean pre-pregnancy BMI and were younger. Indian-born mothers were significantly more likely than their Australian counterparts to be highly educated and to live in socioeconomically disadvantaged (SEIFA) areas. A higher proportion of Indian-born mothers had their spouse/ partners currently living with them, a lower proportion of Indian-born mothers smoked during pregnancy and had three or more children compared to the Australian-born mothers (*p* < 0.05). Regarding language spoke at home, 19.6% of Indian-born mothers spoke English at home. The mean age of infants of Indian-born mothers was (6.5 ± 4.6) months and Australian-born mothers (6.6 ± 5.1) months with a range of 1 – 25 months (data not shown).

**Table 1:** Demographic characteristics of Indian-born mothers and Australian-born mothers living in Australia

Demographic characteristics		Indian-born mothers	Australian-born mothers	*p*-value
		***n* = 501**	***n* = 510**	
		Mean (SD)	Mean (SD)	
Maternal BMI (pre-pregnancy, kg/m2)		23.1 (3.9)	25.6 (5.9)	< 0.001
		Proportion (%)	Proportion (%)	
Maternal age				< 0.001
	15–24 years	6.7	8.4	
	25–29 years	38.1	25.9	
	30–34 years	40.3	35.3	
	35 + years	14.9	30.5	
Higher qualifications				< 0.001
	Tertiary educated	80.8	36.5	
	Diploma/ Certificate	9.9	40.7	
	Year 12 / Year 11 equivalent	9.3	22.8	
Socioeconomic disadvantaged (quintiles)				0.008
	1st quintile (most disadvantaged)	18.3	12.6	
	2nd quintile	14.3	16.1	
	3rd quintile	24.2	21.5	
	4th quintile	23.8	22.4	
	5th quintile (most advantaged)	19.3	27.4	
Income				< 0.001
	$156,000 or more	4.8	10.3	
	$88,400—$155,999	18.9	33.4	
	$52,000—$88,399	31.0	27.6	
	$26,000—$51,999	31.6	17.5	
	$25,999 or below	13.7	11.3	
Smoking status pregnancy				< 0.001
	Smoking	0.4	8.9	
	Not smoking	99.6	91.1	
Parity				< 0.001
	1 child	59.2	38.3	
	2 children	35.3	39.1	
	3 or more children	5.5	22.5	
Spouse living in the house	Yes	96.8	92.2	0.001
	No	3.2	7.8	
Main language spoken English	Yes	19.6	100	< 0.001
	No	80.4	0.0	

Chi-square test calculated significance level of categorical variables and t-test for continuous variables

### Infant feeding practices

Table 2 presents the infant feeding practices of Indian-born mothers and Australian-born mothers. Compared to infants of Australian-born mothers, infants of Indian-born mothers received any breastmilk for longer (3.6 ± 3.9 months vs 6.1 ± 4.0 months, *p* < 0.05), were currently receiving breastmilk (60.9% vs 76.6%, *p* < 0.001). A significantly higher proportion of infants of Indian-born mothers continued to receive breastmilk at or after six months compared to infants of Australian-born mothers (42.9% vs 21.3%, *p* < 0.05). No significant differences were observed between the two groups for age first exposed to formula and ever drunk formula. Nevertheless, the proportion of infants exposed to formula was high in both groups. Both groups introduced solids earlier than recommended six months of age; however infants of Indian-born mothers were older when exposed to soft semisolids or solids compared to infants of Australian-born mothers (5.3 ± 1.6 months vs 4.7 ± 1.1 months, *p* = 0.01),. Similarly, a significantly lower proportion of infants of Indian-born mothers were exposed to soft semisolids or solids before six months compared to infants of Australian-born mothers (56.6% vs 80.1%, *p* < 0.001), thus showing some compliance with Australian infant feeding guidelines. In contrast, infants of Indian-born mothers were exposed to fruit juice, water-based drinks and cow's milk significantly earlier than infants of Australian-born mothers (fruit juice: 7.4 months vs 9.8 months, water-based-drinks: 7.2 months vs 9.6 months, cow’s milk: 9.2 months vs 10.5 months, *p* < 0.05). However, there was no difference between the two groups for exposure to fruit juice before six months (*p* = 0.162). A significantly higher proportion of infants of Indian-born mothers ever had fruit juice compared to infants of Australia-born mothers (31.2% vs 18.5%, *p* < 0.05). No significant differences were found between the groups who had ever drunk cow's milk, toddler milk and soymilk, or ever drunk water-based drinks before six months.

**Table 2:** Infant feeding practices in infants of Indian-born and Australian-born mothers living in Australia

Infant feeding practices	Indian-born mothers		Australian-born mothers		*p*-value
	n	Median (IQR)	n	Median (IQR)	
Age when stopped breastmilk (months)	105	4 .0 (2.0—10.0)	174	2.0 (1.0—5.0)	< 0.001
	**n**	**Mean (SD)**	**n**	**Mean (SD)**	***p*-value**
Age when stopped breastmilk (months)	105	6.1 (4.0)	174	3.6 (3.9)	0.008
Age when drank infant formula product (months)	317	1.7 (2.2)	322	1.6 (2.4)	0.84
Age when first drank cow's milk (months)	71	9.2 (4.2)	58	10.5 (3.1)	< 0.001
Age first drank water (months)	267	3.8 (2.1)	286	3.3 (2.3)	0.08
Age when first drank water-based drinks (months)	85	7.2 (5.1)	52	9.6 (6.1)	0.02
Age when first drank fruit juice (months)	117	7.4 (3.9)	70	9.8 (5.4)	< 0.001
Age when first ate solid, semisolid foods (months)	219	5.3 (1.6)	261	4.7 (1.1)	0.01
	**n**	**Proportion (%)**	**n**	**Proportion (%)**	***p*-value**
Currently receiving breastmilk (yes)	383	76.6	283	60.9	< 0.001
Age when stopped breastmilk (months)					0.007
< 6 months	60	57.1	137	78.7	< 0.001
≥ 6 months	45	42.9	37	21.3	
Ever drunk cow's milk	74	18.8	60	15.1	0.195
Ever drunk formula (yes)	314	80.9	282	79.2	0.622
Ever drunk soymilk (yes)	5	1.3	10	2.5	0.313
Ever drunk any water-based drinks (yes)	86	22.1	58	14.6	0.009
Age introduced water-based drinks (months)					0.224
< 6 months	34	40	14	26.9	0.17
≥ 6 months	51	60	38	73.1	
Ever drunk fruit juice (yes)	122	31.2	74	18.5	< 0.001
Age introduced fruit juice (months)					
< 6 months	43	36.8	18	25.7	0.162
≥ 6 months	74	63.2	52	74.3	
Ever eaten soft, semisolid, solid foods (yes)	217	55.5	261	66.1	< 0.001
Age introduced soft, semisolid, solid foods (months)					
< 6 months	124	56.6	209	80.1	< 0.001
≥ 6 months	95	43.4	52	19.9	

Water-based drinks: cordial, soft drinks, tea (excludes diluted fruit juice and infant formula products). Note: Variation in sample size due to range in age of infants which were possibly too young to have been exposed to these infant feeding practices

  Table 3 presents results from the multiple linear logistic regression models that assessed the influence of ethnicity (Indian-born vs Australian-born) on infant feeding practices. Similar results were revealed from unadjusted and adjusted models. Adjusted models showed that infants of Indian-born mothers were breastfed 2.5 months longer and were exposed to soft, semisolid solid foods 0.7 months later than infants of Australian-born mothers (*p* < 0.001). Likewise, in the adjusted model, infants of Indian-born mothers were 36% less likely ever to have ever had solids and 67% less likely to introduce solids before six months compared to the infants of Australian-born mothers (*p* < 0.001). Infants of Indian-born mothers exposed their infants to fruit juice, water-based drinks and cow’s milk earlier than infants of Australian-born mothers (*p* < 0.05). However, water was introduced significantly later (Beta = 0.41 months, *p* < 0.05) amongst infants of Indian-born mothers compared to their counterparts. Infants of Indian-born mothers were 1.8 times and 2.7 times more likely ever to be exposed to water-based drinks and fruit juice, respectively (*p* < 0.05). However, no differences were observed for the introduction to cow’s milk, formula, soymilk, water, and consumed water-based drinks and fruit juice before six months, age of exposure to formula and also the likelihood of consuming formula amongst the two groups.

**Table 3:** Ethnic differences in infant feeding practices amongst Indian-born and Australian-born mothers in Australia

Variable	Model 1 (unadjusted *n* = 501)			Model 2 (adjusted *n* = 510)		
	B Coeff	95% CI	p-value	B Coeff	95% CI	*p*-value
Age stopped receiving breastmilk (months)						
Indian vs Australian	2.5	1.4, 3.6	< 0.001	2.13	1.23, 3.01	< 0.001
Age when first drank infant formula products (months)						
Indian vs Australian	0.07	- 0.29, 0.43	0.705	0.05	-0.35, 0.44	0.817
Age when first drank cow's milk (months)						
Indian vs Australian	-1.35	-2.64, 0.05	0.042	-1.97	-3.04, -0.89	0.001
Age when first drank water (months)						
Indian vs Australian	0.48	0.12, 0.85	0.009	0.41	-0.02,0.80	0.038
Age when first drank water-based drinks (months)						
Indian vs Australian	-2.4	-4.3, -0.49	0.014	-2.82	-4.01, -1.62	< 0.001
Age when first drank fruit juice (months)						
Indian vs Australian	-2.36	-3.71, -1.0	0.001	-2.66	-3.49, -1.83	< 0.001
Age when first ate soft, semisolid solid foods (months)						
Indian vs Australian	0.65	0.41, 0.90	< 0.001	0.64	0.39, 0.88	< 0.001
**Variable**	**OR**	**95% CI**	***p*-value**	**OR**	**95% CI**	***p*-value**
Infant currently receiving breastmilk (yes)	2.11	1.59, 2.78	< 0.001	2.67	1.53, 3.36	< 0.001
Infant ever had formula (yes)	1.11	0.78, 1.60	0.559	1.39	0.89, 2.16	0.145
Infant ever had cow's milk (yes)	1.3	0.90, 1.89	0.165	1.91	0.94, 3.89	0.076
Infant ever had soymilk (yes)	0.5	0.17, 1.46	0.203	0.77	0.21, 2.86	0.696
Infant ever had water (yes)	0.89	0.66, 1.22	0.478	0.8	0.52, 1.21	0.286
Infant ever had any water-based drinks (yes)	1.63	1.13, 2.36	0.009	1.79	1.06, 3.03	0.029
Infants given water based drinks < 6 months (yes)	1.81	0.85, 3.83	0.122	2.65	0.63,11.12	0.184
Infant ever had fruit juice (yes)	1.94	1.39, 2.69	< 0.001	2.74	1.62, 4.64	< 0.001
Infants given fruit juice < 6 months (yes)	1.68	0.87, 3.23	0.121	1.68	0.87,3.23	0.121
Infant ever had solids (yes)	0.64	0.48, 0.85	0.002	0.30	0.16, 0.56	< 0.001
Infants given solids < 6 months (yes)	0.33	0.22, 0.49	< 0.001	0.33	0.18, 0.58	< 0.001

Water-based drinks: cordial, soft drinks, tea, coffee(excludes fruit juice and infant formula), Model 1: Unadjusted model, Model 2: Adjusted model for maternal age, maternal pre-pregnancy BMI, current infants age, SEIFA, parity, smoking status upon model1

## Discussion

The present study is the first known study to compare infant feeding practices in a large and nationally representative sample of Indian-born mothers and Australian-born mothers living in Australia. These two groups showed significant differences in infant feeding practices.

Overall, Indian-born mothers were more likely than were Australian-born mothers to meet Australian infant feeding guidelines relating to prolonged duration of breastfeeding, higher likelihood of currently breastfeeding, later exposure to water and solids and reduced likelihood of introducing solids before six months. However, infant feeding practices such as exposure to water-based drinks, fruit juice, cow’s milk and formula were found to be suboptimal and fell short of infant feeding guidelines.

### Breastfeeding

Higher breastfeeding rates amongst the Indian-born mothers’ contrasts with other Australian studies where migrant Chinese and Vietnamese mothers living in Australia showed a low breastfeeding rate when the infant was three months of age (36% and 60%) [29, 30]. However, findings from the current study align with those from national and international studies (Australia, UK and the US), where Indian-born immigrant mothers showed higher breastfeeding rates and breastfeeding duration compared to white mothers [16, 22, 31, 32].

The higher breastfeeding rate among Indian mothers may be due to stronger views about breastfeeding as an integral aspect of their cultural heritage and positive influence of social support on breastfeeding practices for Indian immigrant mothers, as shown in a Melbourne study [16, 21, 24, 31, 32]. Consistent with our findings, results from Australian and UK studies have shown that ethnicity predicted the likelihood of an infant currently receiving breastmilk and prolonged breastfeeding compared to white mothers [33, 34]. Similarly, an Australian study that found that Indian-born mothers breastfed their infants for eight weeks longer than Australian-born mothers, thus suggesting that ethnicity is a predictor of breastfeeding practices [35]. In the present study, despite the positive finding, more than half of Indian-born mothers had stopped breastfeeding before six months, suggesting a high proportion not breastfeeding their infants for the first 12 months.

Given the protective effect of breastfeeding through the first 12 months on infant weight status and dose-dependent relationship with obesity, the earlier cessation of breastfeeding observed amongst both the groups, it is likely that infants in both groups are at increased risk of being overweight and obese [36, 37].

### Formula feeding and other fluids

Limited evidence exists on formula feeding practices amongst infants of Indian-born mothers in Australia, and the international evidence is mixed. Although there were no in-between differences groups for formula feeding, a very high proportion (79– 81%) of infants in both the groups consumed formula at two months of age. Formula feeding alone or combined has been associated with higher overweight or obesity risk [38]. These results suggest that the current formula feeding practices of infants of Indian-born and Australian-born mothers may put these infants at higher risk of subsequent overweight or obesity.

The Australian infant feeding guidelines do not recommend cow’s milk, fruit juice or any water-based drinks (tea, coffee, soft drinks, cordials or any other beverages) for infants under 12 months of age [12]. In the present study, a large number of mothers, regardless of ethnicity, did not meet these guidelines. Furthermore, compared to infants of Australian-born mothers, Indian-born mothers in this study introduced fruit juice significantly earlier. These findings are consistent with previous literature where ethnic mothers (Pakistani, Asian immigrant mothers (UK) and Chinese immigrant mothers in Australia) have a higher likelihood of introducing fruit juice sooner than white mothers [39–41]. Moreover, evidence has shown that Hispanic, black and black non-Hispanic infants consumed a significantly higher proportion of sugar-sweetened beverages (SSBs) between 1 – 12 months than white infants [42]. Given the link between SSB consumption and obesity, it is possible that infants of Indian-born mothers are at increased risk of developing obesity and insulin resistance in adulthood [43–46]. A UK cross-sectional study by Ehtisham et al., revealed that compared to white European adolescents, South Asian adolescents (including Indian) were significantly overweight/ obese (12% vs 42%), had significantly higher fasting insulin levels, were less insulin sensitive and had significantly higher total cholesterol levels [46]. The increased risk of diabetes amongst South Asian adolescents may be contributed to acculturation. In a Canadian study, Adjel et al. reported that long-term immigrants (arrived/migrated > 10 years) had significantly greater odds of getting diabetes (OR:2.30, *p* < 0.001) compared to newly arrived immigrants (arrived/migrated < 10 years; OR:1.43, *p* = 0.234) or white Canadian-born adults suggesting a link between acculturation and increased likelihood of suffering from diabetes [47].

Additionally, the current study also showed that one-fifth of infants of Indian-born mothers were introduced to cow's milk by around nine months, which was significantly earlier compared to infants of Australian-born mothers. Early exposure to cow's milk (before 12 months) has been associated with an increased risk of gastrointestinal blood loss, leading to iron deficiency anaemia and an increased risk of developing chronic diseases such as diabetes and renal overload [48]. Early education regarding the harmful effects of early exposure to cows' milk is warranted in Indian-born mothers to prevent associated health risks.

### Introduction to solids

Around 57% of Indian-born mothers and 88% of Australian-born mothers introduced soft, semisolids and solids before six months of age, which doesn't align with Australian infant feeding guidelines [12]. However, Indian-born mothers were less likely to introduce solids before six months compared to Australian-born mothers. This ethnic difference in the solid introduction has been previously reported in some Australian studies where Chinese, Vietnamese and Indian mothers showed a reduced likelihood of introducing solids before four months [39, 49]. Current findings are consistent with previous national and international literature where lower acculturation and positive breastfeeding attitudes amongst Indian-immigrant mothers resulted in the delayed introduction of solids [16, 49]. It is, therefore, possible that Indian-born mothers in this study were less acculturated and had positive attitudes towards breastfeeding, leading to a delayed introduction to solids.

There is limited evidence on the type of foods infants are offered during weaning amongst Indian-born mothers. As shown in international studies, a higher proportion of immigrant mothers from India, Pakistan and Turkey offer sweet foods as introductory foods during weaning, accompanied by a ceremony [40, 50, 51]. Infants from immigrant mothers were also more likely to consume commercial baby foods, chips or roast potatoes, sugar-sweetened drinks and fruit juice compared to white infants [41]. Given this evidence, understanding how infants are fed helps us to understand how to support parents to provide healthy food intakes to promote healthy growth in infants [52]. Future studies examining weaning foods infants are exposed to in Indian immigrant families is warranted.

### Strengths and limitations

The current study contributes to crucial knowledge regarding differences in infant feeding practices of Indian-born mothers compared to their non-immigrant counterparts living in Australia, forming a baseline for future research. A particular strength of this study is that it draws on a large randomly selected sample of nationally representative data on infant feeding practices, thus reducing selection bias and increasing generalisability of the findings to the broader population of Indian-immigrant mothers living in Australia. The study also adjusted for a range of covariates that are linked with infant feeding practices. However, there are study limitations to acknowledge. The Australian National Infant Feeding Survey was conducted ten years ago, but remains the most recent and comprehensive national infant feeding survey in Australia. Given the cross-sectional nature of the data, causality cannot be inferred. Other limitations include the potential of recall bias and social desirability bias, and risks inherent to self-reported questionnaires. Further, the current study did not assess infant anthropometry nor length of acculturation. It will be valuable to include these measures in future studies.

### Future recommendations

Future studies amongst Indian-born mothers are warranted to explore the implications of ethnicity on infant feeding practices such as breastfeeding initiation, pre-lacteal feeding, combination feeding of breastmilk and formula and types of solids introduced. Furthermore, the influence of cultural beliefs, confinement practices, biomedical factors such as mode of delivery, family support from parents, grandparents, friends and health care professionals and acculturation on infant feeding practices amongst this group is warranted. Due to the increased risk of overweight and obesity the Indian-immigrants [3], there is also a need to examine the association between infant feeding practices and child anthropometry; along with the long-term incidence of overweight and obesity in children; and how this compares to children of Australian-born mothers.

## Conclusion

The current study has provided valuable insight into infant feeding practices amongst Indian-born mothers living in Australia. Health-promoting practices of Indian-born mothers included prolonged duration of breastfeeding, later introduction of solids, water and reduced likelihood of introducing solids before six months. However, sub-optimal and potentially obesogenic infant feeding practices such as the early introduction of water-based drinks, fruit juice, cow's milk and formula were evident amongst Indian-born mothers. Given the increase in migration from India alongside the rise in the prevalence of childhood overweight/obesity, it is crucial to set up the children of Indian immigrants for optimal growth and health behaviours for life. These findings help build a picture where to focus resources to support Indian-born mothers to feel confident to desist formula and other sweetened fluids in their infant's early life.

## Data Availability

In 2010–2011 The Australian Institute of Health and Welfare (AIHW) conducted The Australian National Infant Feeding Survey (ANIFS) (25), a large scale, national survey of infant feeding practices and behaviours with infants 0–24 months of age. The dataset used in this study is publicly available via special request form (https://www.ada/edu.au/accessing-data) and with the approval by the data custodian (Australian Data Archive). All data generated during this current study are included in this manuscript.

## Abbreviations

ANIFS: Australian National Infant Feeding Survey
BMI: Body Mass Index
IYCF: Infant and Young Child Feeding Guidelines
SEIFA: Socio-Economic Indexes For Areas
WHO: World Health Organization
